# Transobturator-cable-fixation in pelvic ring injuries with symphyseal disruption – a last resort?

**DOI:** 10.1007/s00068-024-02578-9

**Published:** 2024-08-07

**Authors:** Martin C. Jordan, Richard Wagner, Lukas Hufnagel, Justus Bremer, Maximilian Heilig, Philipp Heilig, Christopher P. Bretherton, Rainer H. Meffert

**Affiliations:** 1 Department of Orthopaedic Trauma, Hand, Plastic and Reconstructive Surgery, University Hospital Würzburg, Oberdürrbacherstraße 6, 97080 Würzburg, Germany; 2 Center of Orthopaedics, Trauma Surgery and Rehabilitation Medicine, University Medicine Greifswald, Fleischmannstr. 8, 17475 Greifswald, Germany; 3 Bone and Joint Health, Blizard Institute, Queen Mary University London, 4 Newark Street, London, E1 UK

**Keywords:** Open-book injury, Osteosynthesis, Pelvic fracture, Pelvic injury, Plating, Symphyseal rupture, Symphyseal disruption, Severly injuried patients, Trauma, Osteosynthesis

## Abstract

**Purpose:**

The role of transobturator-cable-fixation (TOCF) in traumatic symphyseal rupture of the pelvic ring remains unclear. This case series aims to evaluate TOCF in complex and revision cases in pelvic surgery.

**Methods:**

A retrospective analysis of a chronological case series was conducted, studying pelvic fractures stabilized using TOCF between January 2006 and December 2022. The variables considered included age, gender, fracture classification, Injury Severity Score (ISS), Body Mass Index (BMI), trauma mechanism, time to surgery, fixation technique, hospital duration, complications, status on discharge (Glasgow Outcome Scale; GOS), follow-up time and indication for the use of TOCF.

**Results:**

All patients (*N* = 7) were male with a mean age of 64 years and a mean BMI of 29. The mean ISS was 45, with the lowest ISS of 25, indicating that only polytraumatized patients were included. Two anterior-posterior-compression-, four lateral-compression-, and one vertical-shear-pelvic-injury were identified. TOCF was added in six cases to support symphyseal plating and in one case to external fixation. The mean hospital stay was 49 days and the mean follow-up duration was 8.5 months. No complications associated with TOCF were observed during the surgical procedure or follow-up.

**Conclusion:**

TOCF showed no procedure-associated complications and effectively supported symphyseal healing in all cases. The main indications were obesity, poor bone quality in elderly patients, and revision cases. TOCF could be considered as a last treatment option in open-book pelvic injuries where plating or external fixation is at risk to fail.

**Supplementary Information:**

The online version contains supplementary material available at 10.1007/s00068-024-02578-9.

## Introduction

Open reduction and plating represent the preferred treatment of symphyseal disruption in traumatic pelvic ring injuries. The well-established surgical technique involves a direct approach, application of reduction tools to close the symphyseal gap, and plate fixation. The success of this surgical technique relies on a stable anchored implant and patient compliance. Risk factors for early implant failure and re-gapping include poor bone quality, obesity, non-compliance and a high degree of posterior pelvic instability, as in vertical shear injuries [1, 2]. Implant loosening, indicated by screw pull-out is frequently seen in follow-up x-rays and is caused by regular micro-movement of the pubic symphysis. However, in most cases, it has no clinical consequences [3–8]. If implant failure occurs before the pubic symphysis is healed, re-gapping might follow and require revision surgery. This can be seen as a downside of symphyseal plating. Alternative fixation techniques are therefore required. Good procedures such as internal/external fixator or bridge plating have been described in the past. Transobturator cable fixation (TOCF) is another alternative fixation technique, but is not currently used regularly because its biomechanical properties are controversial. Some data suggest promising stability [9–11]. Although reported in the early history of pelvic surgery, it never gained widespread use or acceptance [12, 13]. Hereby, a cable is passed through the obturator foramen, encircling the pubic symphysis and securing the reduction of the joint. It can be used as an addition to symphyseal plating or external fixation. The indications for TOCF are unclear and to our knowledge, there are no other case series in the literature. Due to the lack of clinical data, we present our experience with this rare technique in a chronological case series.

## Methods

We retrospectively analyzed patients with a pelvic ring injury requiring surgery between 2006 and 2022 at a level 1 trauma center. Patients were identified by International Statistical Classification of Diseases and Related Health Problems (ICD), Classification of Interventions and Procedures (OPCS) codes, and manual searches. Classification of the injury and analysis of the surgical procedure was made by clinical examination and a CT scan. Patient records served as follow-up data. All data was anonymized and stored on the clinical data storage system with restricted access. The following variables were investigated: Age at the time of injury, gender, Body Mass Index (BMI), classification (AO and Young & Burgess), Injury Severity Score (ISS), trauma mechanism, time to surgery, fixation technique, length of hospital stay, complications, status on discharge (GOS), and follow-up time. Data collection and analysis was conducted using SPSS (v. 25, IBM, Armonk, USA). A 1.7 mm stainless steel braided cable (Orthopaedic Cable System; Depuy Synthes) was used from the surgical site. A Pfannenstiel incision was used for insertion, with careful anterior preparation to avoid damage to the spermatic cord and retraction of the bladder, similar to a Stoppa approach. The study was carried out in accordance with the ethical standards of the institution and the Helsinki Declaration and its later amendments. Approval was granted by the Ethical Committee of the University of Würzburg (AZ 20,231,212 01; 13.12.2023).

## Results

A total of 932 cases were screened and seven patients with TOCF procedures were selected. The mean ISS was 45 (± 12.6) and the lowest ISS was 25, indicating that only severely injured patient were treated with TOCF. The mean BMI was 30 (± 3.7); however, in two patients the BMI could not be calculated retrospectively. All types of pelvic injuries were included. The injury patterns were Anterior-Posterior Compression (APC)-injury in two cases, Lateral Compression (LC)-injury in four cases and Verti­cal Sheer (VS)-injury in one case. However, the posterior more unstable types such as APC III, LPC II/III or VS were more common. In particular, we observed several highly unstable LC type III injuries according to the Young and Burgess classification. The windswept pelvis in our cases included significant injury to the contralateral hemipelvis with laceration of the anterior sacroiliac ligament. The injury patterns were Anterior-Posterior Compression (APC)-injury in two cases, Lateral Compression (LC)-injury in four cases and Vertical Sheer (VS)-injury in one case. In six cases the TOCF was added to symphyseal plating and in one case to external fixation. The mean hospital stay was 49 days (± 21) and the mean follow-up time was 258 days. The mean Glasgow-Outcome Scale was 3.4 (± 1.0) (Table 1). The cases are summarized as followed:

**Table 1:** Summary of the main findings

Case	Age	Classification	ISS	BMI	Fixation	Ex.Fix.	In-patient days	Indication for TOCF	Complications	Glasgow Outcome Scale on discharge	Follow-up
#1	48	61B3.3d, APC II	57	29	TOCF	yes	61	High risk for implant infection	Surgical site infection	3	2 months
#2	83	61B2.2, LC III	34	28	6-hole plate and TOCF	yes	25	Poor bone quality Senile osteoporosis	no	1	1 month
#3	62	61C2.1d, LC III	66	26	4-hole plate and TOCF	yes	37	Poor bone quality Senile osteoporosis	no	4	1 month
#4	79	61C1.1d, LC III	25	30	6-hole plate and TOCF	yes	18	Poor bone quality Senile osteoporosis	no	4	10 months
#5	68	61C1.1d, LC III	47	37	4-hole and 6-hole plate and TOCF	yes	76	Obesity	Surgical site infection	4	2.5 months
#6	38	61C1.3, APC III	45	-	4-hole plate and TOCF	yes	69	Obesity Loss of reduction with plating only	no	4	27 months
#7	67	61C3.1d, VS	43	-	4-hole plate and TOCF	no	58	Loss of reduction with plating only	no	4	15 months

TOCF: Transobturator cable fixation, ISS: Injury Severity Score, BMI: Body Mass Index, Ex. Fix.: External Fixation

### Case#1 2003176171

**Fig. 1:**
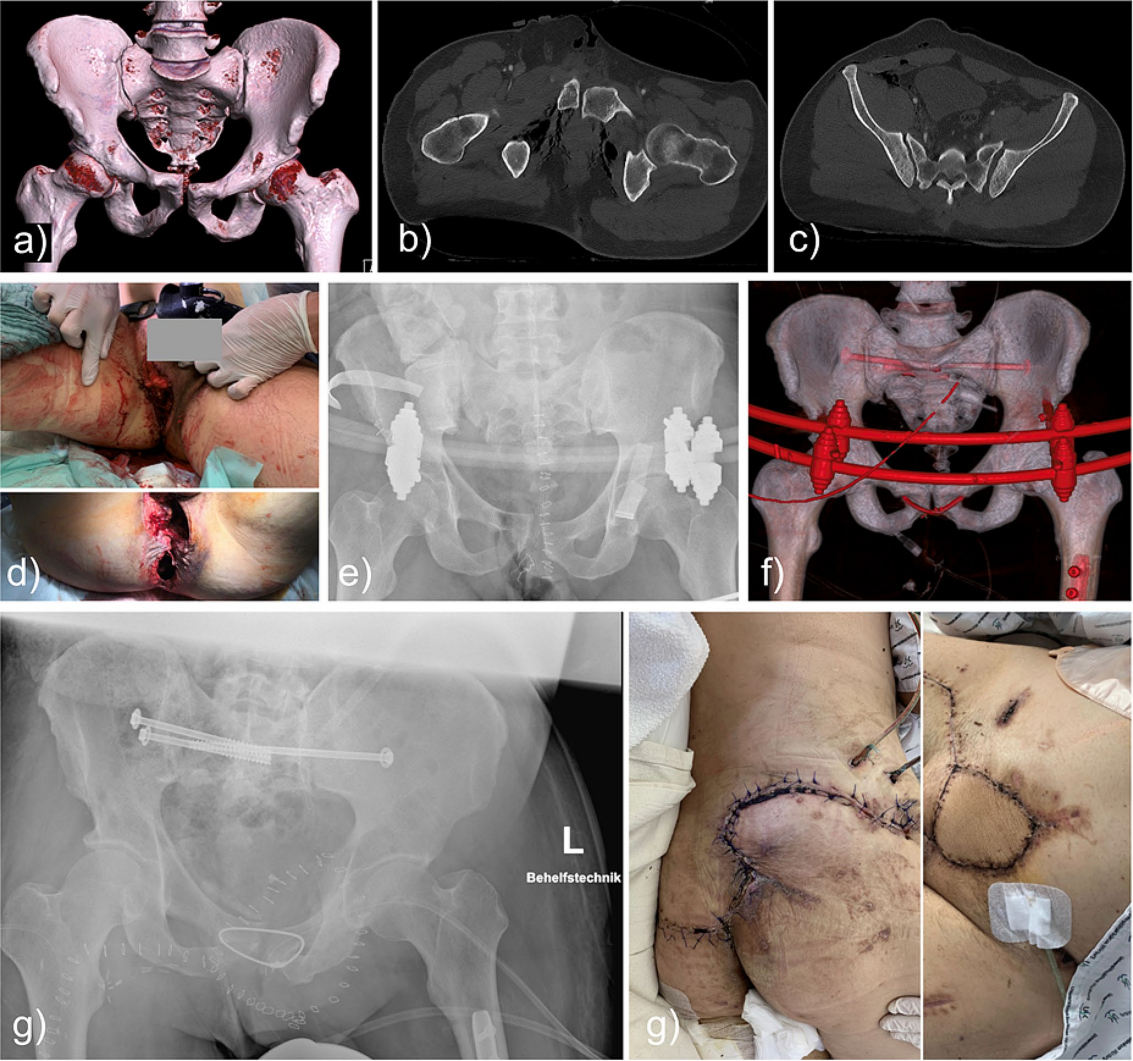
Case#1: **a-c**) Open pelvic injury with disruption of the pubic symphysis and the iliosacral joints on both sites. Severity of the injury may be underestimated in the CT scan due to the pelvic binder. Air within the true pelvis indicates the open injury. **d**) Pubic injury with rupture of the rectum, rupture of the pelvic floor and injury to the urethra. The wound was contaminated with wood. Surgical debridement through a midline incision and external fixation followed. **e**) X-ray of the supraacetabular fixation. **f**) Subsequent infection required multiple surgical wash outs and vacuum wound therapy. TOCF was chosen instead of a plate in combination with external fixation and SI-screws. **g**) Removal of the fixator was necessary because of a pin-tract infection. Finally, plastic surgeons were able to close the wound with flaps

A-48-year old male patient suffered a perineal open pelvic injury with widening of the pubic symphysis and both iliosacral joints (AO-61B3.3d; APC II) caused by a fall into a wood timber conveyor belt (Fig. 1). ISS was 57 demonstrating the severity of the injuries. Contamination, rectum perforation and urethra lesion caused severe infection. Several surgical wound debridements and plastic reconstructions were necessary. In total, 16 surgical interventions were performed before he was discharged after 61 days. Surgical stabilization of the pelvis was performed using sacroiliacal screws (SI-screws) on both sides and an initial supraacetabular external fixator. The reason for TOCF in this case was to avoid bulky implant material within an open suprasymphyseal defect before plastic closure. The cable fixation successfully secured the reduction, allowing the patient to be mobilized. The patient, a foreign guest worker, was lost to follow-up after discharge.

### Case#2 2002813696

An 83-year old male cyclist collided with a motor vehicle and sustained a pelvic ring injury with symphyseal widening and a crescent fracture on the left (AO: 61B2.2, LCII, Day Type II). Multiple injuries resulted in an ISS of 34. On admission, damage control was conducted including supraacetabular external fixation. Due to poor bone quality, early loosening occurred, necessitating internal fixation, carried out with a 6-hole symphyseal plate with additional TOCF because of impaired screw anchoring. The surgical procedure was completed without complications. Several days later, the geriatric patient died from a nosocomial pneumonia in the intensive care unit.

### Case#3 2003340041

**Fig. 2:**
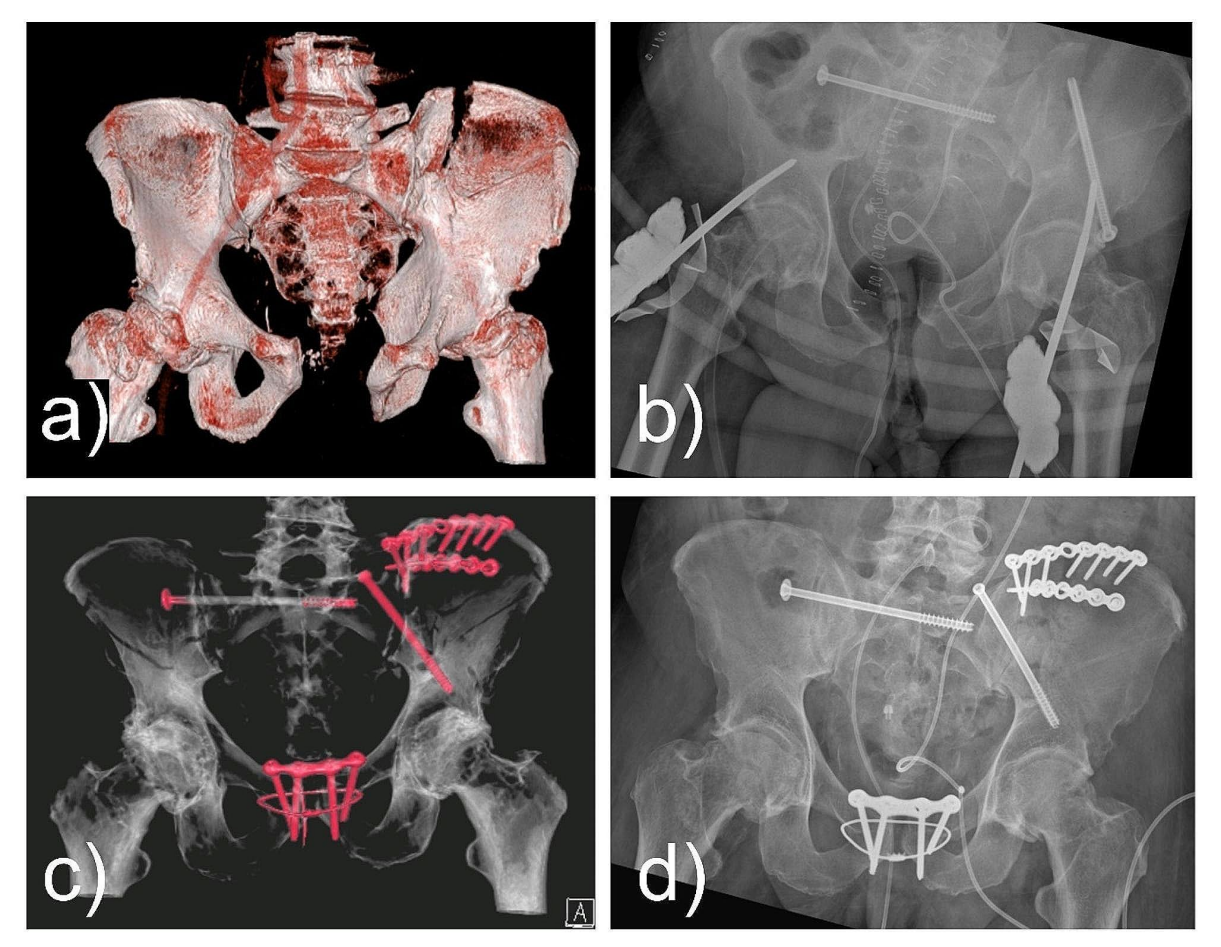
Case#3: **a**) CT scan of a pelvic ring injury with widening of the pubic symphysis, the iliosacral joint and fracture of the ilium (AO: 61C2.1d). Further the patient had a severe kidney laceration with significant hemorrhage and destruction of the ureter. **b**) Emergency treatment was done with external fixation and SI- and supraacetabular screw placement. **c**) Final treatment was done using symphyseal plating with TOCF, plating of the ilium and screw-revision to a LC-screw. **d**) The pelvic ring injury healed but the patient had a prolonged in-hospital stay because of the kidney injury

A 62-year-old male was hit by a heavy pallet and buried underneath, sustaining a pelvic ring injury with widening of the pubic symphysis, the iliosacral joint and a fracture of the ilium (AO: 61C2.1d; LCIII; Day Type II). The ISS was 66. Emergency treatment involved external fixation of the pelvis and a SI-screw (Fig. 2). Urology was involved to treat a rupture of the kidney and ureter. Poor bone quality caused early loosening of the external fixation. Symphyseal plating using a 4-hole plate with additional TOCF and ilium plating was performed. A supraacetabular screw was revised to a posterior-to-anterior lateral-compression-screw. Justification for additional TOCF was to improve the implant strength within an elderly patient. No complications were observed.

### Case#4 2002202055

**Fig. 3:**
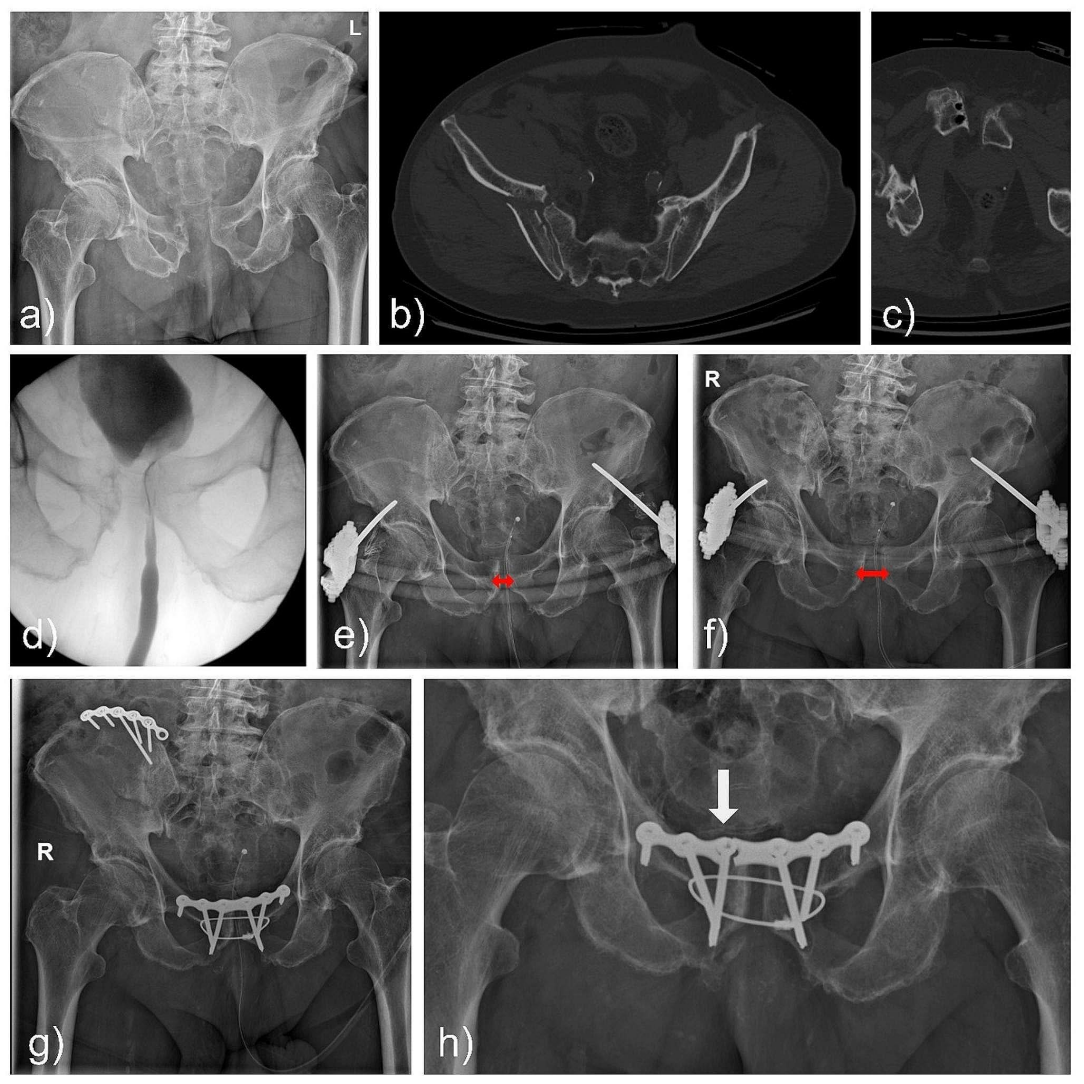
Case#4: **a-c**) X-ray and CT scan of a pelvic ring injury including disruption of the pubic symphysis and fracture of the ileum (AO: 61-C1.1d) in a geriatric patient. Bone cysts within the superior pubic ramus indicate already an impaired bone quality. **d**) Retrograde cystography prior to surgery did not reveal injury of the urinary system. **e-f**) An external fixator was used for emergency treatment, however, loss of reduction with widening of the pubic symphysis was observed within days (red arrows). **g**) Therefore, TOCF with symphyseal plating was performed. In retrospect, iliac wing plating appears to be insufficient for adequate posterior stabilization. **h**) Symphyseal plate breakage was observed during follow-up x-rays (white arrow). The cable remained in place, securing the reduction. This example highlights the benefit of TOCF

A 79-year-old male patient fell from 3 m height and sustained an injury of the pelvic ring including disruption of the pubic symphysis and fracture of the ilium (AO: 61-C1.1d; LC II; Day Type I). The ISS was 25 and routine retrograde cystography was done prior to external fixation. An X-ray of the pelvis after six days revealed significant loss of symphyseal reduction. Consequently, symphyseal plating (6-hole) complemented by TOCF due to impaired bone quality was performed. No intraoperative complications occured and mobilization as well as discharge were as planned. Pelvic x-ray after one year demonstrated plate breakage, which might have caused a recurrence of the symphyseal gapping without TOCF (Fig. 3).

### Case#5 2002611542

**Fig. 4:**
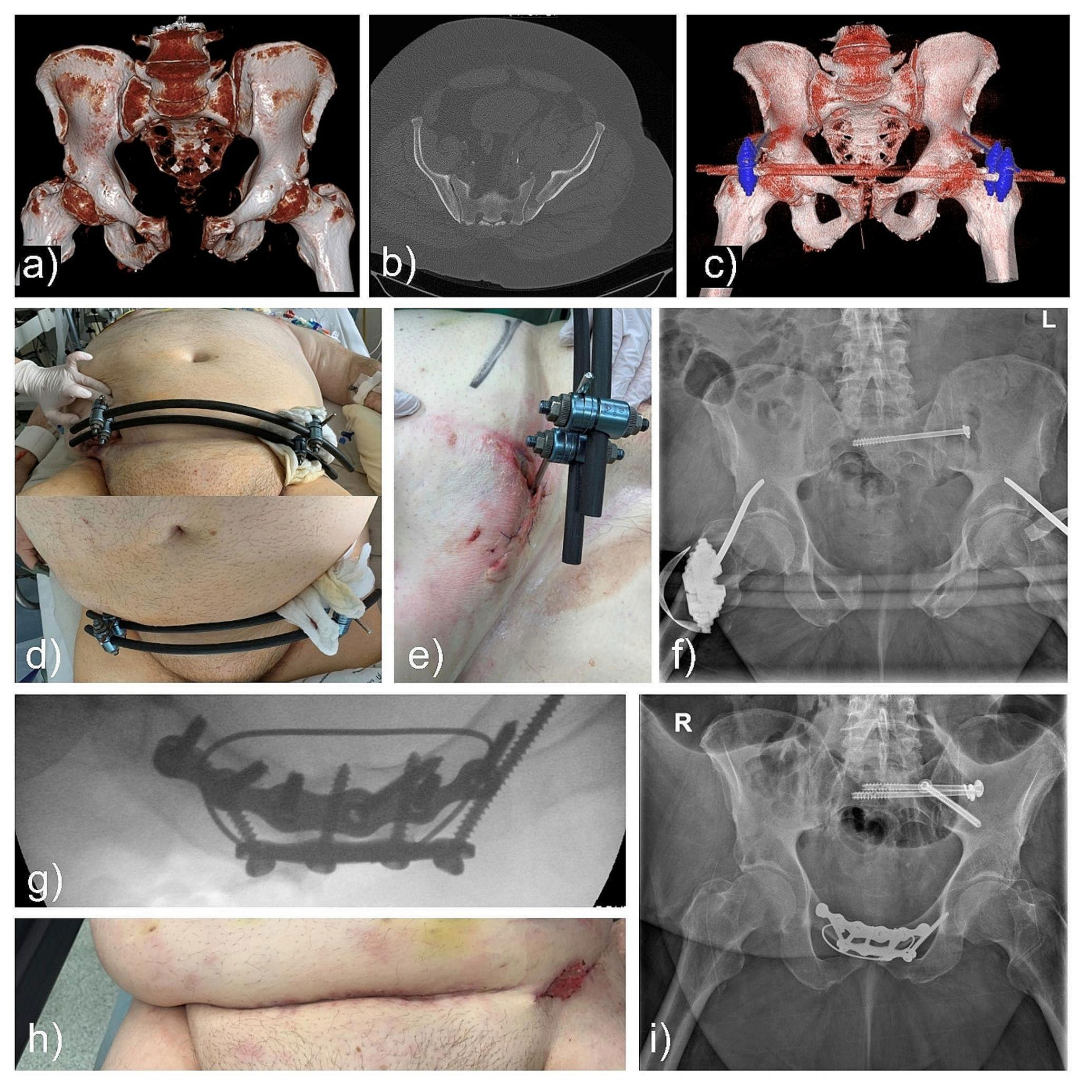
Case#5: **a-c**) An obese patient sustained a pelvic ring injury with symphyseal widening and a unilateral crescent fracture (AO: 61C1.1d). **d-e**) Common problems in these patients are pin-tract infections. **f**) Loss of reduction was observed in the x-ray image days later, before mobilization. **g**) Revision was done with double-plating and TOCF. Unfortunately, the patient had a surgical site infection. **h**) The infection was controlled after various wound debridements. **i**) The pelvic ring remained stable after mobilization

A 68-year-old male patient fell from several stairs and was transferred from another hospital. The extremely obese patient (BMI 37) suffered from a pelvic ring injury with symphyseal widening and a unilateral crescent fracture (AO: 61C1.1d, LC II; Day Type III). External fixation was the initial treatment choice considering the distinct obesity. As loss of symphyseal reduction occurred (Fig. 4), open reduction and fixation with a 6-hole symphyseal plate, an additional 4-hole-anterior-plate and reinforcement with TOCF was necessary. The reason for TOCF was anticipated implant loosening due to the heavy load. The surgical procedure itself was without complications but the patient developed a surgical site infection with a multisensitive staphylococcus epidermidis. Another four surgical wound washouts were necessary, further complicated by a prolonged ICU stay due to respiratory problems. However, the pelvic injury healed and the patient was regularly followed up in the outpatient clinic.

### Case#6 2002492682

A 38-year-old obese motorcyclist suffered a perineal open pelvic ring injury (AO: 61C1.3d, APCIII) with a unilateral complete sacral fracture and symphyseal disruption (ISS 45). A pelvic c-clamp and a symphyseal plate were placed initially. A local infection postponed posterior fixation, which caused symphyseal plate loosening. During revision surgery, TOCF was added to the symphyseal plate revision. Ilio-iliacal rod fixation was chosen for the posterior pelvic ring. The pelvic injury healed under this treatment without further complications and regular X-rays showed screw loosening with an intact TOCF.

#### Case#7 2002500483

A 67-year-old patient suffered a bilateral crescent fracture with pubic symphysis disruption from a fall from a 2 m height (AO: 61C3.1d; Day Type right: III; left: II). Symphyseal fixation using a 4-hole plate was chosen for the first treatment and posterior stabilization was planned for a second, delayed procedure. In the meantime, the symphseal plate loosened, requiring re-symphyseal plating, the addition of TOCF. In combination with the posterior fixation, the injury healed regularly but heterotopic ossification was noted on follow-up radiographs.

## Discussion

The high mean ISS demonstrates that only severely injured patients were treated with TOCF. Although having a limited follow-up, no direct TOCF associated complications were noticed for these complex cases. Importantly, no re-gapping occured in the available follow-up radiographs. However, all patients were male, with no females identified or included in our study. This reflects the unequal gender distribution in the trauma context, with only 30% of polytraumatized patients being female [14].

In our case series, the main indications for TOCF were revision surgery, obesity, and impaired bone quality. TOCF might bring additional stability in combination with symphyseal plating, which has not yet been proven in biomechanical analysis. It might be that TOCF alone, without symphyseal plating provides enough stability but this theory requires further clinical evaluation [10]. In case#1 TOCF was combined with temporary external fixation and yielded a successful result. However, it is important to note that the combination of two techniques presented in all of our cases does not allow conclusions to be drawn about the stability of the TOCF itself. It may even be that the additional effect of TOCF is very limited.

**Fig. 5:**
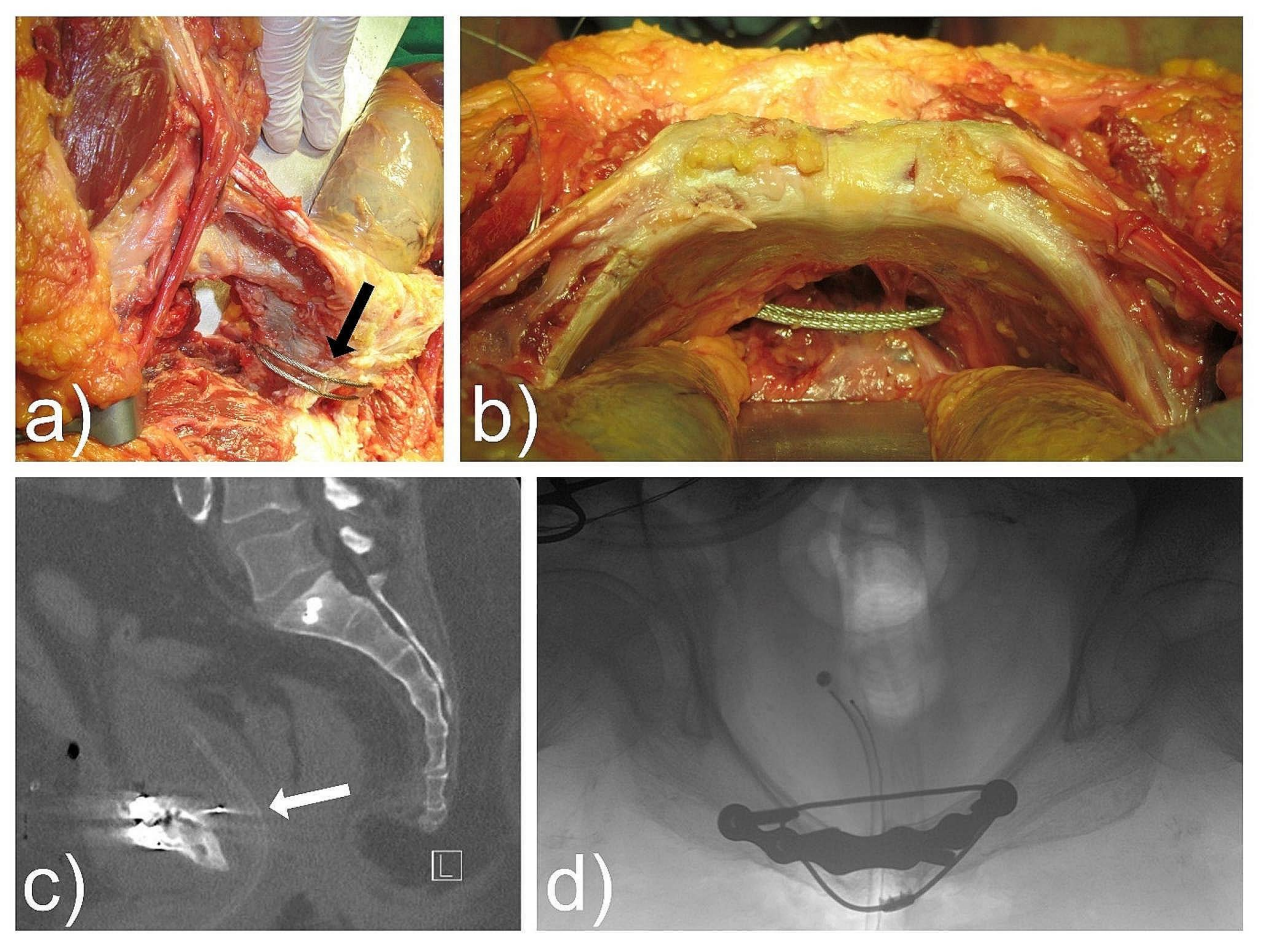
Anatomical considerations: **a**) Demonstrates TOCF in a male cadaver study. Black arrow shows pubic symphysis from anterior. **b**) The cable usually does not cling to the bone and therefore is prominent in the retropubic space (Cave of Retzius). This might be a risk factor causing bladder irritation and lesion. **c**) Demonstrates the close proximity of the bladder. **d**) Shows the prominent cable in an in-let view of the pelvis

Several anatomical structures are at risk during this procedure (Fig. 5). The surgical technique requires mobilization of the bladder, digital identification of the urethra and the inserted urinary catheter, detachment of external and internal muscles at the medial border of the obturator foramen, and passage of the cable under visual control without harming surrounding structures. The spermatic cord is in close proximity and is vulnerable to injury [15, 16]. Structures within the obturator canal are usually on the opposite site of the obturator foramen and can be protected [17]. Nerves and vessels within the Lacuna musculorum and vasorum are more laterally. However, in trauma contexts with symphyseal gapping, anatomical structures might be unexpectedly positioned. Therefore, minimal invasive cable placement without visualization and identification of the mentioned anatomical structures should be avoided. Further, the cable will likely be prominent in the retrosymphyseal space, potentially causing irritation of the bladder.

Contraindications for TOCF are displaced fractures of the pubic ramus, previous surgical interventions with scarring around the bladder, or radiotherapy of the pelvis. Very poor bone quality may also be a contraindication, as the steel cable may cut into the bone during tensioning.

The limitations of this observational report are the following: It is an uncontrolled, anecdotal report of a small group of patients that only have a specific surgical intervention in common. Time of follow-up is limited and some patients were lost early. Despite these limitations, TOCF could still be a viable surgical option in difficult cases where symphyseal plating alone is at risk of failure. This case series could stimulate valuable debate within the surgical community and generate new research questions.

## Conclusion

- TOCF was performed in this case series without intraoperative or early on-set complications.
- TOCF was able to prevent loss of reduction of the pubic symphysis.
- The main indications were impaired bone quality with reduced screw anchorage and obesity with heavy load vectors.
- TOCF was used in addition to symphyseal plating or external fixation.

## Electronic supplementary material

Below is the link to the electronic supplementary material.

Supplementary Material 1

## Data Availability

No datasets were generated or analysed during the current study.
