# Supplementary material for: Transobturator-cable-fixation in pelvic ring injuries with symphyseal disruption – a last resort?
Source: Eur J Trauma Emerg Surg. 2024 Aug 7;50(5):2559–67. doi: 10.1007/s00068-024-02578-9 (PMC11599439; doi:10.1007/s00068-024-02578-9)
Supplement: Supplementary file 1 — Supplementary Material 1 [file 68_2024_2578_MOESM1_ESM.pdf]

Julius-Maximilians-
UNIVERSITÄT
WÜRZBURG

Medizinische Ethikkommission an
der Julius-Maximilians-Universität
Würzburg

Med. Ethikkommission • Josef-Schneider-Str. 4 • 97080 Würzburg

Medizinische Ethikkommission an der JMU
Josef-Schneider-Str. 4, C15
97080 Würzburg

Vorsitzender: Prof. Dr. R. Jahns
Geschäftsführerin: C. Michel
Sachbearbeitung: A. Geßlein, A. Meister,
A. Metzger, S. Schmidt
Telefon 0049 (0)931 31 48315
Telefax 0049 (0)931 31 87520
ethikkommission@uni-wuerzburg.de
www.med.uni-wuerzburg.de/ethik-kommission/startseite

PD Dr. med. Martin Jordan
Klinik und Poliklinik für Unfall-, Hand-, Plastische
und Wiederherstellungschirurgie (Chirurgie II)
ZOM/Oberdürrbacher Str. 6
97080 Würzburg

Würzburg, 13.12.2023/ub

bei Schriftwechsel bitte angeben: **20231212 01**

Retrospektive Datenauswertung
Projekt: Augmentation der Symphysenplatte mittels Kabelcerclage zur Stabilisierung Symphsemsprenung

Sehr geehrter Herr Dr. Jordan,

zu Ihrer Anfrage vom 12.12.2023 zur retrospektiven Auswertung von Patienten- oder Untersuchungsdaten und deren Verwendung in Promotionsarbeiten oder in Publikationen nimmt die Ethikkommission wie folgt Stellung:

Grundsätzlich gilt bei einer Auswertung von bereits vorhandenen, klinikinternen Routinedaten bzw. Daten von individuellen Heilversuchen, dass keine Beratung durch die oder eine Antragstellung bei der Ethikkommission nach geltendem Recht erforderlich ist.

Auf der Grundlage der vorliegenden Informationen bestehen keine grundsätzlichen ethischen oder rechtlichen Bedenken gegen die Auswertung der angeführten Daten für den Zeitraum 01.01.2018 bis 31.12.2023.

Beachten Sie bitte, dass Ergebnisse dieser Auswertung ausschließlich in anonymisierter Form veröffentlicht/publiziert werden dürfen.
Es obliegt dem verantwortlichen Untersucher dafür Sorge zu tragen, dass Auswertung und Publikation mit dem Einverständnis der Klinik-/Institutsleitung unter Beachtung der Richtlinien zur Sicherung guter wissenschaftlicher Praxis erfolgen, dass lediglich Daten in die Auswertung einfließen, die unter Beachtung einschlägiger rechtlicher Vorgaben, als auch berufsethischer Aspekte generiert wurden und geltende Datenschutzbestimmungen eingehalten werden. Zusätzlich verweisen wir auf die einschlägigen Vorgaben zum Umgang mit Patientendaten am UKW.

Mit freundlichen Grüßen

Prof. Dr. med. Roland Jahns
Vorsitzender der Ethikkommission

Ausgefertigt im Auftrag

Datum:
2023.12.13
15:49:05 +01'00'

Uli Baege
Stellv. Geschäftsführer der Ethikkommission
